# Synthesis and Characterization of 3,4-Bis[3(2-azidoethoxy)furazan-4-yl]furoxan (DAeTF): A Novel Low-Melting Insensitive Energetic Material

**DOI:** 10.3390/molecules29194607

**Published:** 2024-09-27

**Authors:** Yang Wu, Yuezhou Liu, Fulei Gao, Bin Chen, Tingting Lu, Yinglei Wang

**Affiliations:** 1 Xi’an Modern Chemistry Research Institute, Xi’an 710065, China; wuy_204@163.com (Y.W.);; 2 State Key Laboratory of Fluorine & Nitrogen Chemicals, Xi’an 710065, China

**Keywords:** energetic compounds, single crystal, insensitive, melt–cast explosive, thermal decomposition mechanism

## Abstract

The synthesis and characterization of low-melting-point insensitive energetic materials are crucial due to their increasing applications in melt–cast explosives. In this work, a furazan-derived energetic compound, 3,4-bis[3(2-azidoethoxy)furazan-4-yl]furoxan (DAeTF), exhibiting insensitive and high-energy characteristics, is rationally designed and synthesized. The structure of DAeTF is characterized by nuclear magnetic resonance spectroscopy, Fourier transform infrared spectroscopy, elemental analysis, mass spectrometry, and single-crystal X-ray diffraction. The thermal properties of DAeTF are investigated using differential scanning calorimetry, in situ FTIR spectroscopy and thermogravimetric-differential scanning calorimetry–Fourier transform infrared–mass spectrometry and thermal decomposition mechanism was elucidated in combination with bond energy calculations. The detonation performance of DAeTF is predicted by the EXPLO5 program. The results indicate that DAeTF has thermal stability (T_d_ = 251.7 °C), high energy level (D = 7270 m/s) and significant insensitivity (IS = 60 J). Additionally, its relatively low melting point (T_m_ = 60.5 °C) facilitates processing and loading. These characteristics indicate that DAeTF is a promising candidate as an insensitive melt–cast explosive in future applications.

## 1. Introduction

Melt-cast explosives represent crucial components of energetic materials due to their ease of processing, high energy density, and superior detonation performance [1,2]. Given their distinct advantages in safety, usability, and damage effectiveness, melt-cast explosives have become indispensable materials in the military field [3,4]. Trinitrotoluene (TNT), the most extensively studied and widely applied melt-cast explosive, has a low melting point (80.6 °C) and superior explosive performance [1,2,5]. However, TNT-based castable explosives exhibit several drawbacks, such as oil exudation during long-term storage, inferior mechanical properties, and higher sensitivity, which render them unsuitable for modern insensitive munition requirements. In the field of melt-cast explosives, the development of insensitive munitions (IMs) has become a dominant trend [6,7].

In recent years, advancements in science and technology have facilitated the development of a series of melt-cast explosives, including 1,3,3-trinitroazetidine (TNAZ) [8,9], 3,4-bis(3-nitrofurazan-4-yl)furoxan (DNTF) [9,10,11], 1-methyl-2,4,5-trinitroimidazole (MTNI) [12], 2,4,6-triazido-1,3,5-triazine (TTA) [13,14], 1-methyl-4,5-dinitro-imidazole (MDNI) [15], 2,4,6-trinitro-3-bromoanisole (TNBA) [16], 5,5′-bis(dimethylene nitrate)-3,3′-biisoxazole (BIOM) [17,18], 3,4-dinitropyrazole (DNP) [19], and 2,4-dinitroanisole (DNAN) [20,21,22,23]. Among them, DNAN, a mainstream insensitive melt-cast explosive, is characterized by low impact and friction sensitivity, compatibility with ammonium perchlorate (AP), and a broad energy adjustment range, positioning it as a melt-cast material with substantial potential for diverse applications. Although DNAN is a promising insensitive melt-cast explosive with the potential to replace TNT, it still faces the challenge of a relatively low energy density. Meanwhile, DNTF, as a mainstream high-energy melt-cast explosive, has garnered considerable attention due to its high energy density. However, its high melting point increases risk during explosive loading, and its relatively high sensitivity limit its further development and application. Therefore, there is a need to actively continue developing new insensitive high-energy melt-cast explosives that strike a balance between insensitivity and high energy density.

Inspired by the structure and performance of the high-energy material DNTF and the excellent low-temperature properties of 1,2-bis(2-azidoethoxy)ethane and 1,1′-Oxybis[2-azidoethane], DNTF and 2-azidoethanol were chosen as starting materials to synthesize the energetic material DAeTF via nucleophilic substitution reaction. The ethoxy groups in the structure impart a lower melting point and insensitive characteristics to the molecule, while the incorporation of azido groups and furazan-furoxan rings maintains a high energy level. The structure was characterized using nuclear magnetic resonance (NMR) spectroscopy, Fourier transform infrared spectroscopy (FTIR), elemental analysis (EA), mass spectrometry (MS) and single-crystal X-ray diffraction. The thermal properties were analyzed using differential scanning calorimetry (DSC), in situ FTIR spectroscopy, and thermogravimetric–differential scanning calorimetry–Fourier transform infrared–mass spectrometry (TG-DSC-FTIR-MS) and the energy level was calculated using EXPLO5 [24]. This work not only broadens and strengthens the energetic materials database but also provides a material foundation for the field of insensitive melt–cast explosives, accelerating their development.

## 2. Results and Discussion

### 2.1. Design and Synthesis

As a mainstream high-energy melt-cast explosive, DNTF exhibits high density and energy levels. However, its high sensitivity and melting point are detrimental to the process safety of melt–cast explosives. In our previous work, their azidoethoxy structure endowed 1,2-bis(2-azidoethoxy)ethane and 1,1′-Oxybis[2-azidoethane] with extremely low glass transition temperatures (T_g_ < −100 °C) and favorable sensitivities (IS = 75.7, 34.3 J) [25,26]. Therefore, to obtain a material with a low melting point, low sensitivity, and excellent detonation performance, DNTF was utilized as the structural framework with an azidoethoxy group introduced, resulting in the formation of an insensitive and high-energy material which is suitable for applications in melt-cast explosive systems.

**Figure 1:**
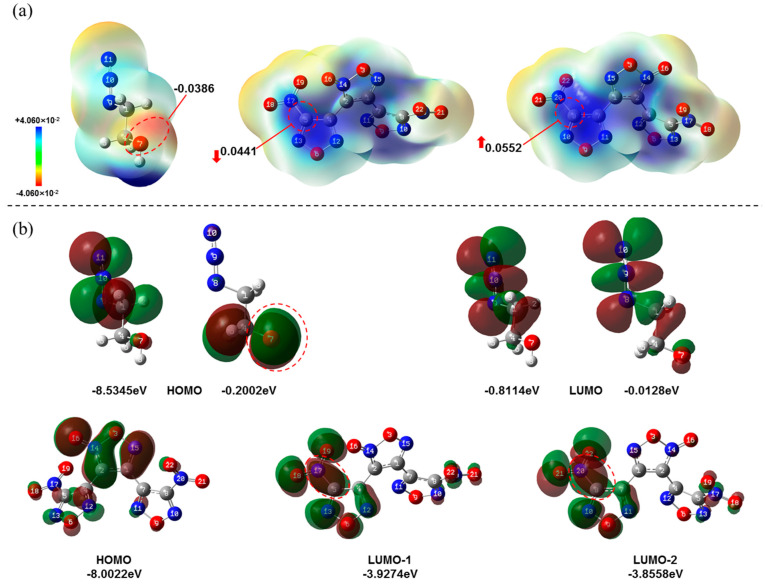
(**a**) Electrostatic potential of 2-azidoethanol and DNTF; (**b**) HOMO and LUMO orbital diagrams of 2-azidoethanol, 2-azethoxyl anion and DNTF.

**Figure 2:**
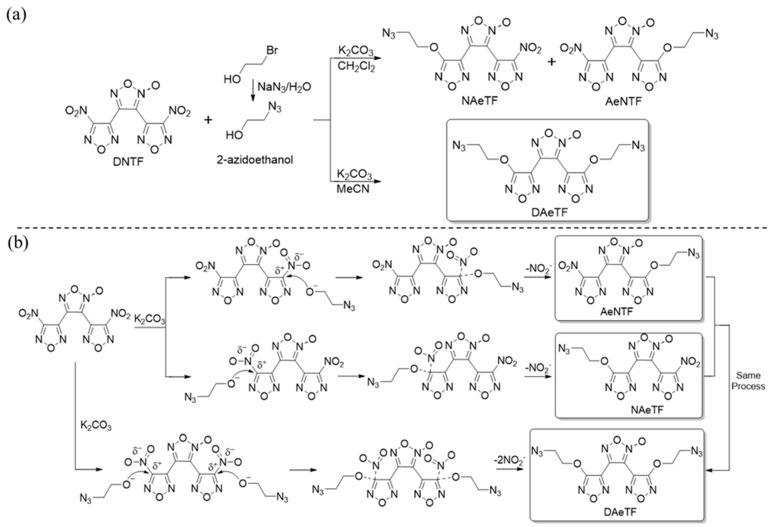
(**a**) Synthesis of NAeTF, AeNTF, and DAeTF. (**b**) Mechanism diagram of nucleophilic substitution reaction of DNTF.

First, DNTF and 2-azidoethanol were selected as the reactants to synthesize DAeTF, and the reaction mechanism was studied through chemical calculations [27,28,29,30,31]. According to the electrostatic potential and molecular orbital diagrams of DNTF and 2-azidoethanol (Figure 1), the HOMO of the oxygen anion at the 7 position of 2-azidoethanol has the smallest orbital energy difference (ΔE = 4.6071, 4.6786 eV) with the LUMO of the carbon atom being attached to the nitro group in DNTF. Additionally, under symmetry-allowed interactions, these orbitals demonstrate maximal overlap [32,33]. The presence of charge shifts in the aromatic nitro group induces the formation of a positive charge center on the carbon atom attached to the nitro group. This suggests that the reaction proceeds through a nucleophilic attack by the oxygen anion of 2-azidoethanol on the nitro-substituted carbon atom in DNTF, resulting in a bimolecular aromatic nucleophilic substitution (Ar-SN2) reaction [34]. Although the furazan ring adheres to the Hückel rule as an aromatic ring, it is a five-membered, six-electron aromatic system consisting of different atoms (C, O, N) which leads to uneven charge distribution. In an SN1 mechanism, the departure of -NO_2_ as the leaving group would result in the formation of a furazan carbon cation, disrupting its aromaticity and making this process less favorable. If the reaction followed an SN1 mechanism, the steric hindrance from the coordinating oxygen would facilitate the departure of -NO_2_ from this side. Since -NO_2_ departure is the rate-determining step in the SN1 mechanism, the product from this side should be more abundant. However, experimental results indicate that the substitution product on the coordinating oxygen side is significantly lower compared to the other side. Therefore, the key step of the reaction involves a nucleophilic attack by the azidoethoxy anion on the furazan ring, leading to the formation of an unstable five-membered transition state intermediate, followed by the departure of the nitro group to complete the reaction. The overall reaction proceeds via an Ar-SN2 mechanism (Figure 2). Additionally, under basic conditions, an intramolecular etherification side reaction of DNTF occurs, as outlined in Figure S1 [35].

The coordinated oxygen in the furoxan ring influences the positive charge density at the substitution site on the furazan ring adjacent to it, which is significantly lower than on the non-coordinated oxygen side [36,37,38]. At low reaction temperatures (37 °C), the products consist primarily of a mixture of single-substituted compounds from both the non-coordinated and coordinated oxygen sides, with a ratio of 5:3 (Figure S8). To achieve a high yield of disubstituted products, the reaction must be carried out at an elevated temperature (6 °C). Consequently, the experiments in dichloromethane and acetonitrile solvents resulted in single-substituted and disubstituted products, respectively.

To reduce side reactions and increase the yield of the desired product, the experimental procedure was modified by first adding 2-azidoethanol and K_2_CO_3_ to the solvent and allowing them to react for 30 min. This initial step ensures a sufficient concentration of the azidoethoxy anion in the reaction system. DNTF was then added to the reaction mixture. This approach significantly accelerates the rate of the primary Ar-SN2 reaction while minimizing side reactions, resulting in an increase in product yield from 37% with the one-time feeding method to 62%.

### 2.2. Single-Crystal X-ray Diffraction

**Figure 3:**
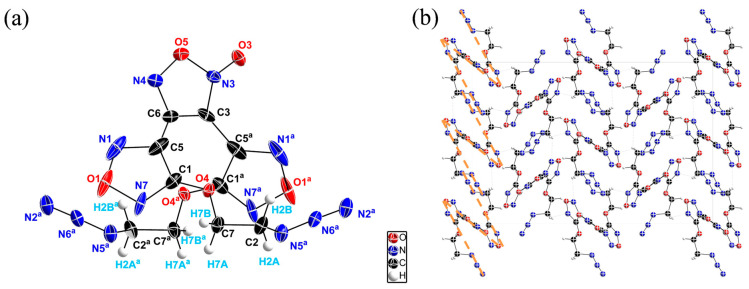
(**a**) Single-crystal structure of DAeTF. (**b**) Packing structure of DAeTF along the c axis.

DAeTF single crystals were obtained through slow solvent evaporation (CCDC: 237789822) [39]. The molecular structure and unit cell packing of the DAeTF crystal are illustrated in Figure 3. Measurements were conducted at 90 K using Mo Kα radiation (λ = 0.71073) to minimize thermal vibrations and achieve precise results. A total of 18,895 diffraction points were collected, ranging from 5.74° to 52.74°, with 1658 independent reflections (R_int_ = 0.0406, R_sigma_ = 0.0265). The final refinement yielded an R factor of R_1_ = 0.0381 and wR_2_ = 0.1027 for I ≥ 2σ(I) and R_1_ = 0.0444 and wR_2_ = 0.1063 for all data, demonstrating high accuracy in the structural refinement. Additionally, the maximum positive electron density peak and the most significant negative electron density hole were found to be 0.73 and −0.69 e Å^3^, respectively, further confirming the precision of the structural resolution.

The crystal structure analysis revealed that DAeTF crystallizes in an orthorhombic system (Fdd2 space group, a = 24.0119(7) Å, b = 16.0697(5) Å, 8.3796(4) Å) with the molecular formula C_10_H_8_N_12_O_6_. The crystal exhibits a density of 1.612 g cm^−3^, and there are eight molecular units per unit cell (Z = 8, V = 3233.40(19) Å^3^).

As shown in Figure 3a, the C1-O4 bond length in the furazan ring (1.2412 Å) is significantly shorter than a typical single bond, and the dihedral angle between N1-C5-C1-O4 is 0.44°, indicating a well-conjugated system within the furazan ether structure. Moreover, the three rings in the DAeTF molecule lie on three distinct planes, with a dihedral angle of 37.56° between the furoxan ring and the furazan ring and a dihedral angle of 75.12° between the two furazan rings (Figure 3a). Furthermore, the bond lengths in the three rings are consistent with standard furazan ring and furoxan ring lengths, suggesting that each of these rings forms a stable conjugated system, adopting a chair-like conformation in space. This molecular packing is similar to that observed in DNTF [16].

As shown in Figure 3b, it was observed that the spatial extension of the ethyl ether chain causes the DAeTF molecule to adopt a zig-zag arrangement in space, with π–π interactions forming between the azido groups of adjacent molecules. Additionally, the ethyl groups lead to complex intermolecular weak hydrogen bonding interactions (C–H···N(O)) [40,41], with numerous hydrogen bonds forming an extensive interconnected network that links the molecules into a cohesive whole (Figure S1).

Overall, the furazan ether forms a stable conjugated system, while the zig-zag molecular structure resulting from the linear extension of the ethyl ether chain facilitates more intermolecular interactions. This is evidenced by the π–π interactions between the azido groups and the numerous intermolecular weak hydrogen bonding interactions (C–H···N(O)). These stable conjugated systems and the orderly intermolecular interactions provide DAeTF with enhanced stability.

### 2.3. Thermodynamic Performance Analysis

In the study of energetic materials, understanding their thermal behavior is crucial for evaluating their stability and performance. Therefore, we conducted a comprehensive thermodynamic analysis of DAeTF, focusing on its melting point, thermal decomposition temperature, and decomposition kinetics, and further elucidated its thermal decomposition mechanism through bond energy calculations. Ultimately, this research provides valuable data to support the application of DAeTF [42,43,44,45].

#### 2.3.1. Thermal Stability

**Figure 4:**
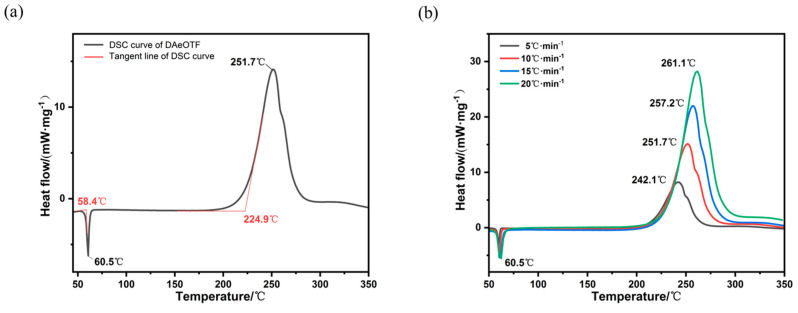
(**a**) DSC curve of DAeTF at a heating rate of 10 K·min^−1^. (**b**) Stacked DSC curves of DAeTF at different heating rates.

The thermal stability of DAeTF was analyzed by DSC. The thermal behavior of DAeTF shows two distinct stages (Figure 4a). The first stage corresponds to a sharp endothermic peak associated with the melting process, while the second stage features an exothermic peak associated with thermal decomposition. The melting of DAeTF begins at 58.4 °C, with a melting peak temperature of 60.5 °C and a melting enthalpy of −77.26 J·g^−1^. The compound undergoes intense exothermic decomposition starting at 224.9 °C, with a decomposition peak temperature of 251.7 °C. The total heat released during the exothermic stage is 2749 J·g^−1^. Due to the introduction of the azidoethoxy group, the melting point of DAeTF has decreased to 60.5 °C, significantly lower than that of DNTF (110 °C). Moreover, the extensive weak hydrogen-bonding network formed between DAeTF molecules enables DAeTF to maintain thermal stability even at temperatures exceeding 100 °C above its melting point, indicating its suitability for safe melt-casting applications.

To determine the thermal decomposition kinetic parameters of DAeTF [46,47], DSC experiments were performed at heating rates of 5, 10, 15, and 20 °C·min^−1^ under a nitrogen atmosphere. The decomposition peak temperatures of DAeTF at these heating rates were recorded as 242.1, 251.7, 257.2, and 261.1 °C, respectively (Figure 4b).

The thermal decomposition kinetics of DAeTF were investigated using both the Kissinger and Ozawa methods. The activation energies calculated from these methods were 158.01 kJ·mol^−1^ and 158.55 kJ·mol^−1^, respectively, showing close agreement and high accuracy, with linear correlation coefficients (R^2^) of 0.9997. Additionally, thermodynamic parameters such as activation enthalpy (∆H), Gibbs free energy (∆G) and activation entropy (∆S) were determined using Equations (S4)–(S6), yielding values of 136.86 kJ·mol^−1^, 154.5 kJ·mol^−1^ and 41.98 J·K^−1^·mol^−1^, respectively. The critical temperature of thermal explosion ($T_{b}$), a key factor in evaluating the thermal stability and safety of energetic materials, was calculated using the Equation (S8). $T_{b}$ was found to be 156.97 °C based on an initial peak decomposition temperature ($T_{P0}$) of 147.24 °C and an activation energy ($E_{0}$) of 158.01 kJ·mol^−1^. These results suggest that DAeTF exhibits excellent thermal stability, making it a viable candidate for practical applications in high-safety environments.

#### 2.3.2. Thermal Decomposition Mechanism

Thermal decomposition behavior is critical in determining the safety and performance of energetic materials. Understanding detailed thermal decomposition mechanism is crucial for the thorough evaluation and application of DAeTF. Therefore, the thermal decomposition mechanism of DAeTF was analyzed using a combination of in situ FTIR spectroscopy, TG-DSC-FTIR-MS analysis, and bond energy calculations [48,49,50,51].

**Figure 5:**
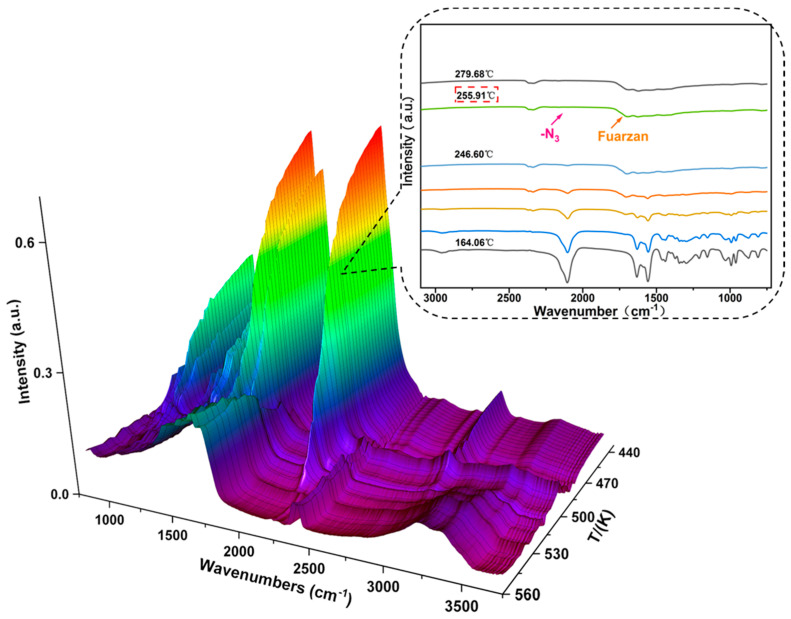
3D image of in-situ FTIR thermal decomposition of DAeTF.

The characteristic infrared absorption peaks of DAeTF begin to weaken at 164.06 °C, indicating the onset of its decomposition process. By the time the temperature reaches 255.91 °C, the infrared absorption peaks corresponding to the functional groups of DAeTF disappear completely (Figure 5).

**Figure 6:**
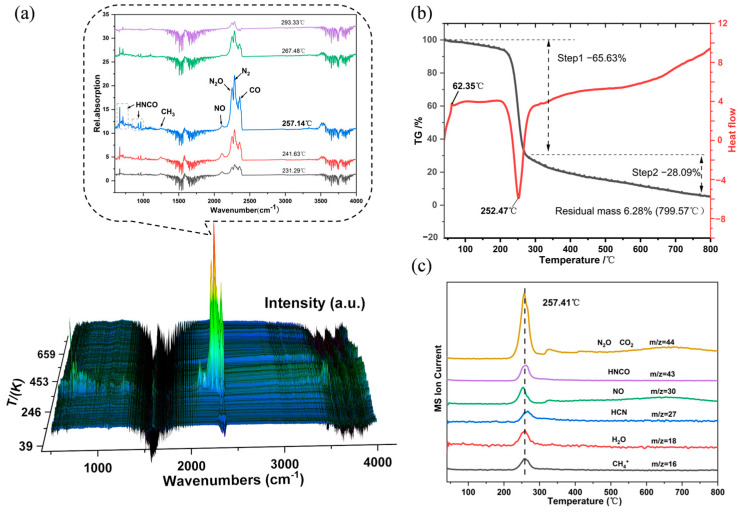
(**a**) Infrared spectrum of decomposition gaseous products of DAeTF. (**b**) TG-DSC curves of DAeTF (TG in black, DSC in red). (**c**) Fragment ion intensity diagram of DAeTF.

The thermal decomposition of DAeTF mainly occurs in two stages. The first stage involves the rapid decomposition near the decomposition peak temperature (252.47 °C), with a mass loss of approximately 65.63%. The second stage, which involves a mass loss of 28.09%, is a continuous and slow decomposition process occurring after the decomposition peak temperature. When the temperature reaches 799.57 °C, the final carbon residue accounts for about 6.28% of the original mass (Figure 6b).

The 3D FTIR absorption spectra are shown in Figure 6a. The infrared characteristic peaks correspond to the vibrational signatures of gas-phase molecules, serving as a fingerprint for identifying gaseous chemical species. At 257.14 °C, the FTIR spectrum exhibits maximum absorption intensity. When the infrared characteristic peaks of DAeTF gaseous products at 257.14 °C were compared with standard gas-phase data from the HITRAN2016 [52] spectroscopy database, strong characteristic peaks for N_2_, CO and N_2_O were identified in the range of 2000–2500 cm^−1^. The peak at 2180 cm^−1^ was attributed to N_2_O, while the peaks between 800 and 2500 cm^−1^ corresponded to HNCO. A weak CH_3_ peak was also observed at 1250 cm^−1^.

As shown in Figure 6c, the ion intensity–temperature curve indicates that the fragmentation products reach full accumulation at 257.37 °C. Due to the interference of the *m*/*z* = 28 ion signal under a nitrogen atmosphere, clear separation of the signal was challenging. However, based on the decomposition products of nitrogen-rich energetic compounds and the infrared spectra of gaseous products, the presence of N_2_ among the gaseous products was confirmed. The CH_3_ fragment likely captures an additional H⁺ to form CH_4_, due to the presence of many H⁺ fragments in the mass spectrometer. Ultimately, the primary oxidation products of DAeTF during thermal decomposition were determined to be CO and N_2_O, with the main nitrogenous products being N_2_ and N_2_O, and the primary hydrogenated products being CH_3_ and a small amount of HNCO.

To gain deeper insights into the thermal decomposition mechanism of DAeTF, the bond dissociation energies (BDEs) of various bonds in DAeTF were calculated using the Gaussian 16 W. The C3–N3 bonds (282.35 kJ·mol^−1^) and N3–O5 bonds (189.48 kJ·mol^−1^) within the furoxan ring were identified as having the lowest bond energies, marking them as the "weak links" in the molecule. Consequently, the furoxan ring in the DAeTF molecule is the first to undergo cleavage, followed by the subsequent breaking of the C–C bond, leading to the formation of two molecules: cyanooxy and azidoethoxy-substituted furazan. The furazan ring is a stable conjugated system with relatively high bond energies; therefore, the initial bond cleavage during heating occurs in the alkyl chain external to the furazan ring.

**Figure 7:**
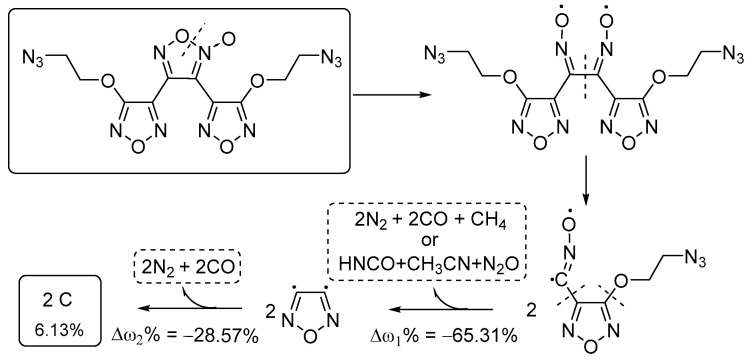
The main thermal decomposition mechanism of DAeTF.

The N2(a)–N6(a) bond (710.02 kJ·mol^−1^) and the N2a–N6a bond (703.36 kJ·mol^−1^) in the azido group have relatively high bond energies, so the azido group primarily decomposes to produce N_2_ gas. The cleavage of the furazan ring and the alkyl chain external to the furazan ring occurs during the same rapid decomposition stage, generating gaseous products: CH_3_, CO, N_2_, CH_3_CN, HNCO, and N_2_O. These gaseous products are also consistent with the results from the infrared spectra. The mass loss at this stage (Δω_1_ = 65.31%) is consistent with the first stage of mass loss observed in the TG-DSC analysis. As the temperature continues to rise, the remaining furazan rings in the molecule cleave further, leading to additional reactions that produce N_2_ and CO. The observed mass loss (Δω_2_ = 28.57%) correlates with the second stage of gradual mass loss observed in the TG-DSC analysis. The final residue is carbonaceous, accounting for approximately ω = 6.13% (Figure 7).

### 2.4. Physicochemical and Detonation Properties

To investigate the physicochemical and detonation properties of DAeTF, its impact sensitivity was measured using a calibrated BAM drop-hammer sensitivity tester. The drop hammer weighed 10 kg, and the explosive sample weighed 50 mg. The tests were conducted by varying the drop height in each trial. The detonation velocity and detonation pressure of DAeTF were calculated using the EXPLO5 [25] and compared with the performance of TNT, DNAN, and DNTF. The results are summarized in Table 1.

The results indicate that DAeTF outperforms the typical insensitive melt-cast explosive DNAN in both density and detonation velocity, with a detonation velocity of 7270 m·s^−1^, which is slightly better than TNT (6970 m·s^−1^). Due to the introduction of ethyl groups, an extensive hydrogen-bonding crosslinked network is formed between DAeTF molecules, while the azido groups establish π–π interactions with the furazan ring. These structural features provide DAeTF with significant sensitivity advantages over TNT, DNAN, and DNTF, solidifying its position as an exceptional insensitive energetic material. The introduction of the azidoethoxy group reduced the sensitivity and the melting point and improved thermal stability. This combination of superior thermal performance and sensitivity, along with energy levels exceeding those of TNT, makes DAeTF an ideal choice as a liquid-phase carrier in melt-cast explosives.

## 3. Materials and Methods

General caution: Although we have experienced no explosion accidents in synthesis and characterization of these materials, proper protective measures should be adopted.

### 3.1. Reagents and Instruments

**Reagents**: 2-bromoethanol, sodium azide, anhydrous potassium carbonate, sodium tungstate dihydrate, 30% hydrogen peroxide solution, dichloromethane, and acetonitrile all of analytical grade, were purchased from Beijing Inokai Science and Technology Co., Ltd, Beijing, China. DNTF is obtained from the Xi’an Modern Chemistry Research Institute.

**Instruments**: AV 500 superconducting nuclear magnetic resonance spectrometer, Bruker, Fällanden, Switzerland; NEXUS 870 Fourier transform infrared spectrometer, Nicolet, Glendale, WI, USA; Vario-EL-3 elemental analyzer, EXEMENTAR, Langenselbold, Germany; LC-2010A high-performance liquid chromatography system, Shimadzu, Japan; DSC 204 differential scanning calorimeter, NETZSCH, Hanau, Germany; Thermo Scientific Q Exactive, Waltham, MA, USA; NETZSCH STA449F3 + FTIR Nicolet iS20 + NETZSCH-QMS 403, Germany; Rigaku XtaLAB Synergy R/S diffractometer, Tokyo, Japan.

### 3.2. Computational Methods

- Electrostatic potential (ESP) and Molecular Orbital Calculations

Geometric optimizations of 2-azidoethanol and DNTF were carried out using Gaussian 16 W [54] using density functional theory (DFT), employing the B3LYP/6-31g (d,p) basis set, and vibrational analysis of the optimized structures revealed no imaginary frequencies, suggesting that the structures reside at minima on their respective potential energy surfaces. Subsequently, the electrostatic potential and molecular orbitals of 2-azidoethanol and DNTF were calculated and analyzed using the B3LYP/6-31+g (d,p) basis set.

- Bond Energy Calculations

The energies of different radical fragments of the DAeTF molecule were calculated using DFT with the uB3LYP/6-31g (d,p) basis set through the Gaussian 16 W. The energy differences between each fragment and the molecule were determined using Equation S1, leading to the determination of the average bond energy within the DAeTF. All calculated values are consistent with standard data and fall within a reasonable range [55].

### 3.3. Synthetic Methods

- Synthesis of 2-Azidoethanol

2-Azidoethanol was synthesized according to the reported work [56]. Yield: 39.08%; HPLC purity: 97.68%.

^1^H NMR (DMSO-*d*6, 500 MHz), δ: 5.00 (t, 1H, -OH, *J* = 5 Hz), 3.58 (q, 2H, -CH_2_, *J* = 5 Hz), 3.27 (t, 2H, -CH_2_, *J* = 5 Hz); ^13^C NMR (DMSO-*d*6, 125 MHz), δ: 60.62 (-O-CH_2_-), 53.34 (-CH_2_-N_3_); FTIR (KBr), ῦ/cm^−1^: 3376 (-OH), 2937, 2881 (-CH_2_-), 2101 (-N_3_), 1297, 1066 (C-O-).

- Synthesis of NAeTF and AeNTF

At room temperature, dichloromethane (50.0 mL), potassium carbonate (1.38 g, 0.010 mol), and 2-azidoethanol (2.17 g, 0.025 mol) were added sequentially to a 50 mL three-necked flask equipped with a thermometer, stirrer, and reflux condenser. The mixture was gradually heated to 37 °C and stirred for 30 min. Then, DNTF (3.12 g, 0.010 mol) was added to the reaction system, and the mixture was stirred at 37 °C for an additional 3–4 h. After the reaction was complete, the mixture was cooled to room temperature, washed three times with 150 mL of deionized water, and concentrated under reduced pressure to obtain a pale yellow viscous liquid (2.96 g), with a yield of 84.1%.

^1^H NMR (DMSO-*d*6, 500 MHz), δ: 4.61–4.59 (t, 2H, CH_2_, *J* = 5 Hz), 4.41–4.39 (t, 2H, CH_2_, *J* = 5 Hz), 3.76–3.74 (t, 2H, CH_2_, *J* = 5 Hz), 3.58–3.56 (t, 2H, CH_2_, *J* = 5 Hz). ^13^C NMR (DMSO-*d*6, 125 MHz), δ: 163.35, 163.29 (C-O of furazan), 160.39 (C-NO_2_ of furazan), 142.22, 140.34, 137.67, 137.01 (C of furazan), 144.89, 105.82, 102.62 (C of furoxan), 72.86, 72.68 (O-CH_2_), 49.52, 49.30 (CH_2_-N_3_); FTIR (KBr), ῦ/cm^−1^: 3384, 3326, 3219 (-NH_2_), 2943, 2857 (-CH_2_-), 2110 (-N_3_), 1635, 1595, 1558, 1460, 1344, 1285 (furazan, furoxan), 1033, 994 (C-O-). Anal. Calcd. for C_8_N_10_O_7_H_4_: C 27.28, H 1.14, N 39.77; Found: C 27.49, H 1.15, N 39.81.

- Synthesis of DAeTF

At room temperature, acetonitrile (20.0 mL), potassium carbonate (1.38 g, 0.010 mol), and 2-azidoethanol (2.17 g, 0.025 mol) were added sequentially to a 50 mL three-necked flask equipped with a thermometer, stirrer, and reflux condenser. The mixture was gradually heated to 60 °C and stirred for 30 min. Then, 3,4-bis(3-nitrofurazan-4-yl)furoxan (3.12 g, 0.010 mol) was dissolved in 10 mL of acetonitrile, and the solution was added dropwise to the reaction system. The mixture was stirred at 60 °C for an additional 3–4 h. After the reaction was complete, the mixture was cooled to room temperature, and the reaction mixture was diluted with 300 mL of deionized water. The product was extracted three times with 40 mL of dichloromethane, and the combined organic phase was washed three times with 150 mL of deionized water and concentrated under reduced pressure [57]. The product was recrystallized from CCl_4_ to obtain a white solid (2.46 g), with a yield of 62.6%.

^1^H NMR (DMSO-*d*6, 500 MHz), δ: 4.57–4.56(t, 2H, O-CH_2_, *J* = 5 Hz), 4.49–4.47 (t, 2H, O-CH_2_, *J* = 5 Hz), 3.71–3.69(t, 2H, CH_2_-N_3_, *J* = 5 Hz), 3.59–3.57 (t, 2H, CH_2_-N_3_, *J* = 5 Hz); ^13^C NMR (DMSO-*d*6, 125 MHz), δ: 163.68, 163.50 (C-O of furazan), 144.01, 103.96 (C of furoxan), 136.85, 134.30 (C of furazan), 72.63, 72.57 (O-CH_2_), 49.54 (CH_2_-N_3_). FTIR (KBr), ῦ/cm^−1^: 2970, 2939 (-CH_2_-), 2158, 2118 (-N_3_), 1633, 1594, 1552, 1480, 1328 (furazan, furoxan), 1010, 990 (-O-). Anal. Calcd. for C_10_N_12_O_6_H_8_ (%): C 30.62, H 2.06, N 42.85; Found: C 30.65, H 2.26, N 43.07. MS (EI) for C_10_H_8_N_12_O_6_ ([M + Cl]^−^) calcd 427.03728, 428.04068, 429.03432, found 427.04481, 428.04816, 429.04160.

### 3.4. Single-Crystal Preparation Method of DAeTF

DAeTF (100 mg) was added to a solution of petroleum ether and ethyl acetate (6 mL) prepared at a 5:1 ratio, heated to 35 °C. Then the solution was hot-filtered to remove any insoluble solids. The filtrate was placed in a test tube and left undisturbed at room temperature for 5 days. Following careful screening, a single DAeTF crystal measuring 0.19 × 0.08 × 0.06 mm^3^ was selected to ensure the acquisition of high-quality diffraction data during the X-ray diffraction analysis.

### 3.5. Thermal Performance Experimental Methods

- Differential scanning calorimetry experiments

The DSC experiment was conducted under atmospheric pressure, with the sample in an aluminum crucible and an empty crucible as the reference. The analysis was performed at a flow rate of 50 mL·min^−1^, with a sample weight of 0.68 mg, in a nitrogen atmosphere, at heating rates of 5, 10, 15, and 20 °C·min^−1^.

- In situ FTIR experiments

A finely ground DAeTF sample (0.7 mg) was thoroughly mixed with 150 mg of dried potassium bromide (KBr) powder. After thorough grinding, the mixture was pressed into a pellet approximately 13 mm in diameter and 1 mm thick. The uniformly translucent pellet was placed in an open, windowless variable-temperature cell for analysis. The temperature ramp rate of the reaction cell was set at 10 °C·min^−1^, with a detection temperature range of 25 °C to 465 °C. The infrared spectroscopy resolution was 4 cm^−1^, with a scan rate of 7.5 files per minute and 8 scans per file. The detector used was DTGS.

- TG-DSC-FTIR-MS experiments

The reaction vessel was first heated to 308 K, then approximately 5 mg samples were loaded into an open aluminum pan and heated to 1073 K at 10 K min^−1^ under a nitrogen flow at 20 mL min^−1^. The spectra of the evolution of gas fragments during the thermal decomposition of DAeTF were recorded by an FTIR spectrophotometer in real-time tracking mode with a scan range of 4000 cm^−1^ to 500 cm^-1^ at a resolution of 2 cm^−1^. The *m*/*z* for gas-phase fragments evolution during DAeTF decomposition was recorded by the mass spectrometer conducting the dynamic scanning in the *m*/*z* range from 1 to 100.

## 4. Conclusions

In this work, we designed and synthesized 3,4-bis[3-(2-azidoethoxy)furazan-4-yl]furoxan (DAeTF), investigated the mechanism of the Ar-SN2 reaction, optimized the reaction conditions, and established an efficient synthetic route for the disubstituted product. The structure of DAeTF was characterized by FTIR, NMR, EA and MS. Single crystals of DAeTF were obtained by slow evaporation of the solvent, and single-crystal X-ray diffraction analysis revealed that DAeTF crystallizes in the orthorhombic crystal system with space group Fdd2 (Z = 8) and density of 1.612 g·cm^−3^. The three rings in the molecule are located on three different planes, forming a chair-like conformation with dihedral angles of 37.56° and 75.12°. Additionally, DAeTF molecules exhibit a zig-zag structure, with intermolecular π–π interactions and hydrogen bonding conferring excellent stability. The thermal properties of DAeTF were analyzed using DSC, in situ infrared spectroscopy, TG-DSC-FTIR-MS and bond energy calculations. The results indicated T_m_ = 60.5 °C, T_d_ = 251.7 °C, ΔH = 136.86 kJ·mol^−1^, ΔG = 154.5 kJ·mol^−1^, ΔS = 41.98 J·K^−1^·mol^−1^ and $T_{b}$ = 156.97 °C. The thermal decomposition process occurs in two stages: rapid decomposition (Δω_1_ = 28.57%) and slower decomposition (Δω_2_ = 28.57%), with carbonaceous residue accounting for ω = 6.13%. The primary gaseous products are CO, N_2_, N_2_O, CH_4_, CH_3_CN, and HNCO. The sensitivity of DAeTF was measured using the BAM drop hammer test (IS = 60 J) and its detonation performance was calculated using EXPLO 5 (D = 7270 m·s^−1^). In conclusion, compared with the typical insensitive melt-cast explosive carrier DNAN, DAeTF exhibits superior performance, including higher energy levels, lower sensitivity, and a lower melting point. Its excellent safety and energy make it a promising candidate for use as the next generation of insensitive high-energy melt-cast explosive carriers.

## Figures and Tables

**Table 1:** Physicochemical parameters and detonation performance of TNT, DNAN, DNTF, and DAeTF.

	*ρ* ^1^ (g·cm^−3^)	*D* ^2^ (m·s^−1^)	*T_m_* ^3^ (°C)	*T_d_* ^4^ (°C)	*IS* ^5^ (J)
TNT [2,5,53]	1.654	6970	80.8	200.9	15
DNAN [3,22,54]	1.544	5320	94.6	236.7	50
DNTF [3,11,12]	1.937	8930	110	275	25
DAeTF	1.612	7270	60.5	251.7	60

^1^ Density; ^2^ detonation velocity; ^3^ melting point; ^4^ thermal decomposition temperature; ^5^ impact sensitivity.

## Data Availability

The data presented in this study are available in the paper.

## Supplementary Materials

The following supporting information can be downloaded at: https://www.mdpi.com/1420-3049/29/19/4607/s1. Figure S1: The Side reaction mechanism of DNTF; Figure S2: The intermolecular hydrogen bond diagram of DAeTF. Tables S1–S7: Crystal data of DAeTF; Table S8: Molecular bond energy data of DAeTF; Table S9: Thermodynamic parameters of DAeTF; Figure S3: In-situ FTIR spectra of DAeTF; Figures S4–S6: FTIR and NMR spectra of 2-azidoethanol; Figures S7–S9: FTIR and NMR spectra of the mixed liquid of NAeTF and AeNTF; Figure S10–S13: FTIR, NMR and mass spectra of DAeTF.
