# Supplementary material for: Synthesis and Characterization of 3,4-Bis[3(2-azidoethoxy)furazan-4-yl]furoxan (DAeTF): A Novel Low-Melting Insensitive Energetic Material
Source: Molecules. 2024 Sep 27;29(19):4607. doi: 10.3390/molecules29194607 (PMC11605241; doi:10.3390/molecules29194607)
Supplement: Supplementary file 1 [file molecules-29-04607-s001.zip › molecules-3225560-supplementary.pdf]

Supporting Information

Synthesis and Characterization of 3,4-Bis[3(2-azidoethoxy)furazan-4-yl]furoxan (DAeTF): A Novel Low-melting Insensitive Energetic Material

Yang Wu [1], Yuezhou Liu [1], Fulei Gao [1], Bin Chen [1], Tingting Lu [1] and Yinglei Wang [1], [2], [*]

1 Xi'an Modern Chemistry Research Institute, Xi'an 710065, China; wuy_204@163.com (Yang Wu.)

2 State Key Laboratory of Fluorine & Nitrogen Chemicals, Xi'an 710065, China

* Correspondence: wangyl204@163.com

1. Mechanism of side reaction 1

2. Single crystal data of DAeTF 2

3. Molecular bond energy data of DAeTF 6

4. Thermal decomposition kinetics of DAeTF 7

5. In-situ FTIR spectrum of DAeTF 8

6. FTIR and NMR spectra of 2-azidoethanol 9

7. FTIR and NMR spectra of NAeTF, AeNTF 11

8. FTIR, NMR and mass spectra of DAeTF 13

References 16

1. Mechanism of side reaction[1]

Under alkaline conditions, electron displacement occurs in the nitro group of the DNTF, leading to the formation of a dipole molecule and the generation of a C-N-O three-membered ring intermediate. Subsequently, cleavage of the C-N bond leads to the formation of a nitrous acid structure. Meanwhile, due to the weaker N-O bond in the dipole molecule, the negatively charged oxygen atom attacks the positively charged carbon atom of another molecule. This ultimately results in the elimination of N_2O_3 and the formation of a furazan ether structure.

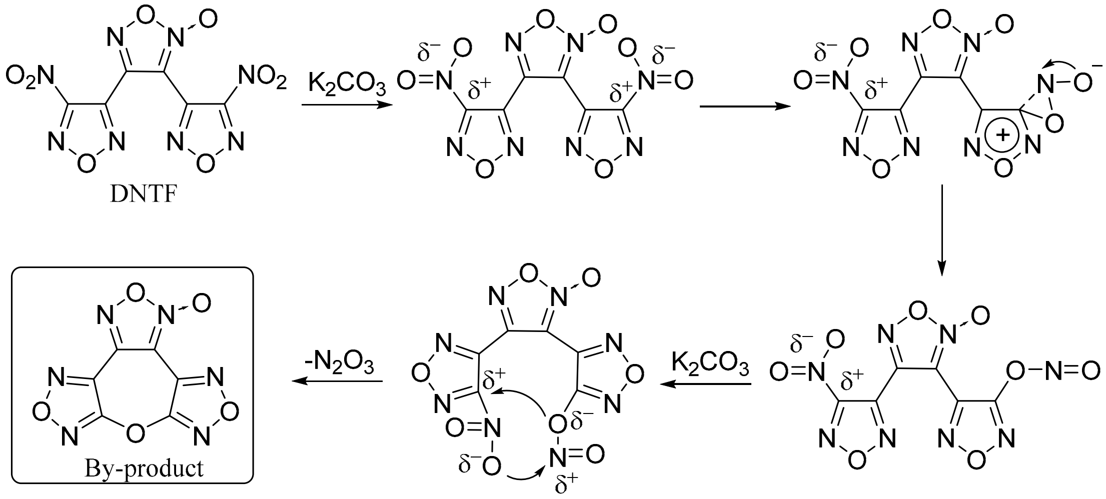

Figure S1. Mechanism diagram of side reaction.

2. Single Crystal data of DAeTF

Table S1. Crystal data and structure refinement for DAeTF.

	DAeTF
Empirical formula	$C_{10}H_8N_{12}O_6$
Formula weight	392.28
Temperature/K	90(15)
Crystal system	orthorhombic
Space group	Fdd2
a/Å	24.0119(7)
b/Å	16.0697(5)
c/Å	8.3796(4)
α/°	90
β/°	90
γ/°	90
Volume/Å^3	3233.40(19)
Z	8
ρ_{calc}g/cm^3	1.612
μ/mm^{-1}	0.136
F(000)	1600.0
Crystal size/mm^3	0.19 × 0.08 × 0.06
Radiation	Mo Kα (λ = 0.71073)
2Θ range for data collection/°	5.74 to 52.738
Index ranges	$-30 \leq h \leq 30$, $-20 \leq k \leq 20$, $-10 \leq l \leq 10$
Reflections collected	18895
Independent reflections	1658 [R_{int} = 0.0483, R_{sigma} = 0.0149]
Data/restraints/parameters	1658/82/145
Goodness-of-fit on F^2	1.068
Final R indexes [I>=2σ (I)]	R_1 = 0.0911, wR_2 = 0.2345
Final R indexes [all data]	R_1 = 0.0916, wR_2 = 0.2349
Largest diff. peak/hole / e Å^{-3}	0.73/-0.69
CCDC number	237789822

Table S2. Fractional atomic coordinates (×10[4]) and equivalent isotropic displacement parameters (Å2×10[3]) for DAeTF.

Atom	x	y	z	U (eq)
O3	569(5)	-540(7)	13199(16)	46(3)
O4	549.3(19)	627(3)	7745(6)	35.9(11)
O5	-194(5)	278(8)	13244(14)	42(2)
N2	-430(3)	3129(4)	7166(11)	55.0(19)
N3	223(6)	-201(9)	12405(14)	38(2)
N4	-547(5)	638(8)	12148(15)	38(2)
N5	25(3)	1959(4)	6089(10)	47.3(16)
N6	-191(3)	2583(4)	6676(9)	41.1(14)
N7	1026(2)	-619(3)	7134(6)	60(2)
C1	687(2)	-95(2)	8093(7)	46.0(19)
C5	542(2)	-542(3)	9495(6)	64(2)
N1	792(3)	-1341(3)	9404(7)	86(3)
O1	1090(2)	-1389(2)	7944(8)	82(2)
C2	630(3)	1881(5)	6326(11)	44.9(19)
C3	121(7)	-111(13)	10839(15)	44(4)
C6	-351(7)	403(10)	10732(16)	35(3)
C7	792(3)	998(4)	6327(10)	42.8(17)

U_{eq} is defined as 1/3 of the trace of the orthogonalised U_{IJ} tensor.

Table S3. Bond lengths for DAeTF.

Atom	Atom	Length/Å	Atom	Atom	Length/Å
O3	N3	1.196(18)	N5	C2	1.471(10)
O4	C1	1.241(5)	N7	C1	1.4200
O4	C7	1.452(8)	N7	O1	1.4200
O5	N3	1.445(15)	C1	C5	1.4200
O5	N4	1.377(18)	C5	N1	1.4200
N2	N6	1.126(10)	C5	C3	1.663(19)
N3	C3	1.342(12)	N1	O1	1.4200
N4	C6	1.332(12)	C2	C7	1.471(10)
N5	N6	1.231(9)	C3	C6	1.41(3)

Table S4. Bond angles for DAeTF.

Atom	Atom	Atom	Angle/°	Atom	Atom	Atom	Angle/°
C1	O4	C7	118.0(5)	C1	C5	C3	119.9(7)
N4	O5	N3	109.0(9)	N1	C5	C1	108.0
O3	N3	O5	117.0(12)	N1	C5	C3	132.0(7)
O3	N3	C3	136.0(17)	C5	N1	O1	108.0
C3	N3	O5	107.0(13)	N7	O1	N1	108.0
C6	N4	O5	104.9(13)	C7	C2	N5	110.1(6)
N6	N5	C2	115.6(7)	N3	C3	C5	120.4(14)
N2	N6	N5	174.3(8)	N3	C3	C6	105.8(15)
C1	N7	O1	108.0	C6	C3	C5	133.7(11)
O4	C1	N7	125.0(4)	N4	C6	C3	113.3(14)
O4	C1	C5	127.0(4)	O4	C7	C2	106.9(6)
N7	C1	C5	108.0				

Table S5. Torsion angles for DAeTF.

A	B	C	D	Angle/°	A	B	C	D	Angle/°
O3	N3	C3	C5	0(3)	C1	O4	C7	C2	-171.6(6)
O3	N3	C3	C6	-177.3(16)	C1	N7	O1	N1	0.0
O4	C1	C5	N1	-179.6(6)	C1	C5	N1	O1	0.0
O4	C1	C5	C3	3.1(9)	C1	C5	C3	N3	-139.3(13)
O5	N3	C3	C5	176.9(11)	C1	C5	C3	C6	38(2)
O5	N3	C3	C6	-0.7(17)	C5	N1	O1	N7	0.0
O5	N4	C6	C3	0.5(15)	C5	C3	C6	N4	-177.0(16)
N3	O5	N4	C6	-0.9(14)	N1	C5	C3	N3	44(2)
N3	C3	C6	N4	0.2(18)	N1	C5	C3	C6	-139.1(14)
N4	O5	N3	O3	178.4(13)	O1	N7	C1	O4	179.6(6)
N4	O5	N3	C3	1.1(16)	O1	N7	C1	C5	0.0
N5	C2	C7	O4	-67.0(9)	C3	C5	N1	O1	176.9(9)
N6	N5	C2	C7	152.0(7)	C7	O4	C1	N7	-6.0(8)
N7	C1	C5	N1	0.0	C7	O4	C1	C5	173.5(5)
N7	C1	C5	C3	-177.4(8)					

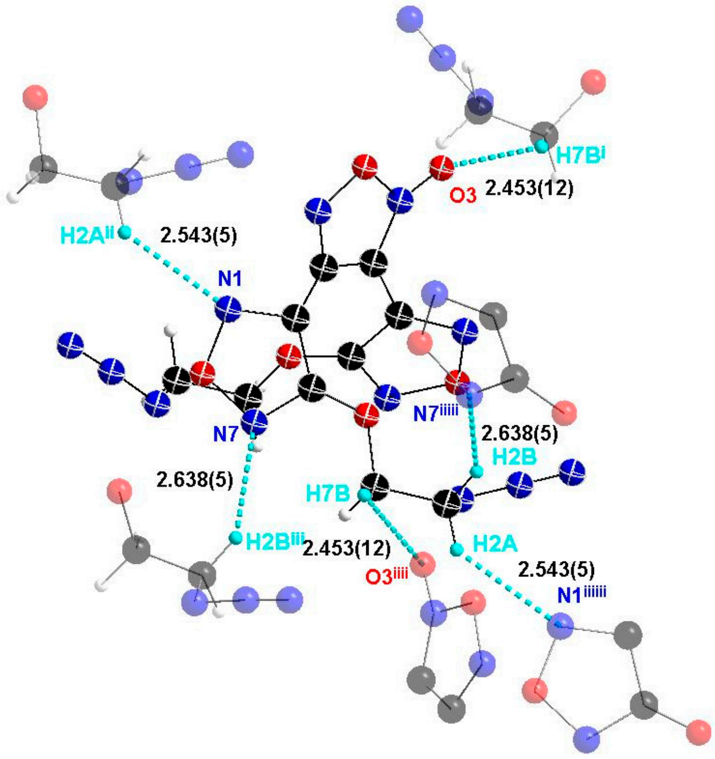

Figure S2. Intermolecular hydrogen bonding of DAeTF (dash lines indicated hydrogen bond interactions).

Table S6. Hydrogen bonds (Å, °) in DAeTF.

D-H···A^{z}	d(D-H)	d(H···A)	d(D···A)	<(DHA)
C2—H2A···N1^{i}	0.9903	2.5428	3.3028	133.424
C2—H2B···N7^{ii}	0.9896	2.6378	3.4338	137.575
C7—H7B···O3^{iii}	0.9905	2.4534	3.1854	130.347
C2a—H2Aa···N1^{i}	0.9903	2.5428	3.3028	133.424
C2a—H2Ba···N7^{ii}	0.9896	2.6378	3.4338	137.575
C7a—H7Ba···O3^{iii}	0.9905	2.4534	3.1854	130.347

^{z} D=donor; A=acceptor. Symmetry code: ^{i} x, 1/2+y,-1/2+z; ^{ii} 1/4-x, 1/4+y, 1/4+z; ^{iii} x, 1/2+y,-1/2+z; ^{iiii} x, 1/2+y, -1/2+z.

Table S7. Atomic occupancy for DAeTF.

Atom	*Occupancy*	Atom	*Occupancy*	Atom	*Occupancy*
O3	0.5	O5	0.5	N3	0.5
N4	0.5	C3	0.5	C6	0.5

3. Molecular bond energy data of DAeTF

$$BDE(A-B) = E(A) + E(B) - E(DAeTF) \quad \text{(S1)}$$

where A and B are the respective radical fragments of the DAeTF molecule.

Table S8. Calculated bond energy of DAeTF.

Bond	BDE[1] (kJ/mol)）	Bond	BDE[1] (kJ/mol)）
N2-N6	710.02	N2a-N6a	703.36
N6-N5	223.11	N6a-N5a	230.81
N5-C2	318.59	N5a-C2a	314.18
C2-C7	399.96	C2a-C7a	435.16
C7-O4	342.58	C7a-O4 a	341.81
O4-C1	443.53	O4a-C1a	448.78
C1-C5	348.78	C1a-C5a	341.45
C1-N7	486.69	C1a-N7a	503.12
C5-N1	579.35	C5a-N1a	553.73
N1-O1	367.83	N1a-O1a	361.87
O1-N7	351.26	O1a-N7a	339.06
C6-C5	529.90	C3-C5a	545.79
C6-N4	457.06	C3-N3	282.35
N4-O5	483.71	N3-O5	189.48
O3-N3	536.91	C3-C6	429.68

[1] Bond energy.

4. Thermal decomposition kinetics of DAeTF

Kissinger Equation:

$$ln\left(\frac{\beta}{T_P^2}\right) = ln\frac{AR}{E} - \frac{E}{RT_p} \quad \text{(S2)}$$

where: β is the heating rate (K·min), T_p is the peak decomposition temperature (K), A is the pre-exponential factor (min^{-1}), R is the universal gas constant (J·(mol·K)$^{-1}$) and E is the activation energy (kJ·mol^{-1}).

Ozawa Equation:

$$log\beta = C - 0.4567\frac{E}{RT_p} \quad \text{(S3)}$$

where: C = log[AE/RG(α)]-2.315 and $G(\alpha)$ is the integral of the conversion function.

The activation energies of DAeTF calculated using the Kissinger and Ozawa methods were 158.01 kJ·mol^{-1} and 158.55 kJ·mol^{-1}, respectively, which are in close agreement. The linear correlation coefficients (R^2) for both methods were 0.9997, indicating excellent accuracy.

$$Ae^{\left(\frac{E}{RT_p}\right)} = \frac{kT_p}{h}e^{\left(-\frac{\Delta G}{RT_p}\right)} \quad \text{(S4)}$$

$$\Delta H = E - RT_P \quad \text{(S5)}$$

$$\Delta G = \Delta H - RT_P\Delta S \quad \text{(S6)}$$

$$T_{P,i} = T_{P0} + m\beta_i + n\beta_i^2 \quad i = 1{\sim}4 \quad \text{(S7)}$$

where: k is the Boltzmann constant (J·K^{-1}) and h is the Planck constant (J·s). β_i is the ith heating rate (K·min^{-1}), $T_{P,i}$ is the peak temperature of thermal decomposition at the heating rate β_i (K), T_{P0} is the peak temperature of thermal decomposition reaction at β→0 (K), m, n is the fitting coefficient.

Using Equations (S4-S6), the activation enthalpy (ΔH), activation Gibbs free energy (ΔG) and activation entropy (ΔS) for the thermal decomposition reaction were determined to be 136.86 kJ·mol^{-1}, 154.5 kJ·mol^{-1} and 41.98 J·K^{-1}·mol^{-1}, respectively.

$$T_b = \frac{E_0 - \sqrt{E_0^2 - 4E_0^2RT_{P0}}}{2R} \quad \text{(S8)}$$

where: E_0 represents the activation energy of the thermal decomposition reaction as β→0 in kJ·mol^{-1}.

The kinetic parameters were then calculated using the Kissinger equation (Equation S2) and the Ozawa equation (Equation S3). These results are summarized in Table S9.

The critical temperature of thermal explosion (T_b), an important parameter for evaluating the thermal stability and safety of energetic materials, was determined using Equation (S8). With T_{P0} at 420.39 K and E_0 at 158.01 kJ·mol^{-1}, T_b was found to be 430.12 K. The results indicated that the excellent thermal stability of DAeTF confers high safety and promising potential for practical applications.

Table S9. Thermodynamic parameters of DAeTF.

β/ (K·min^{-1})	T_p/(K)	Kissinger method			Ozawa method		T_b/(K)	ΔH/(kJ·mol^{-1})	ΔG/(kJ·mol^{-1})	ΔS/(J·K^{-1}·mol^{-1})
		E/ (kJ·mol^{-1})	log(A/ mol^{-1})	R^2	E/(kJ·mol^{-1})	R^2				
5	242.1	158.01	15.57	0.9997	158.55	0.9997	430.12	136.86	154.51	41.98
10	251.7									
15	257.2									
20	261.1									

5. In-situ FTIR spectrum of DAeTF

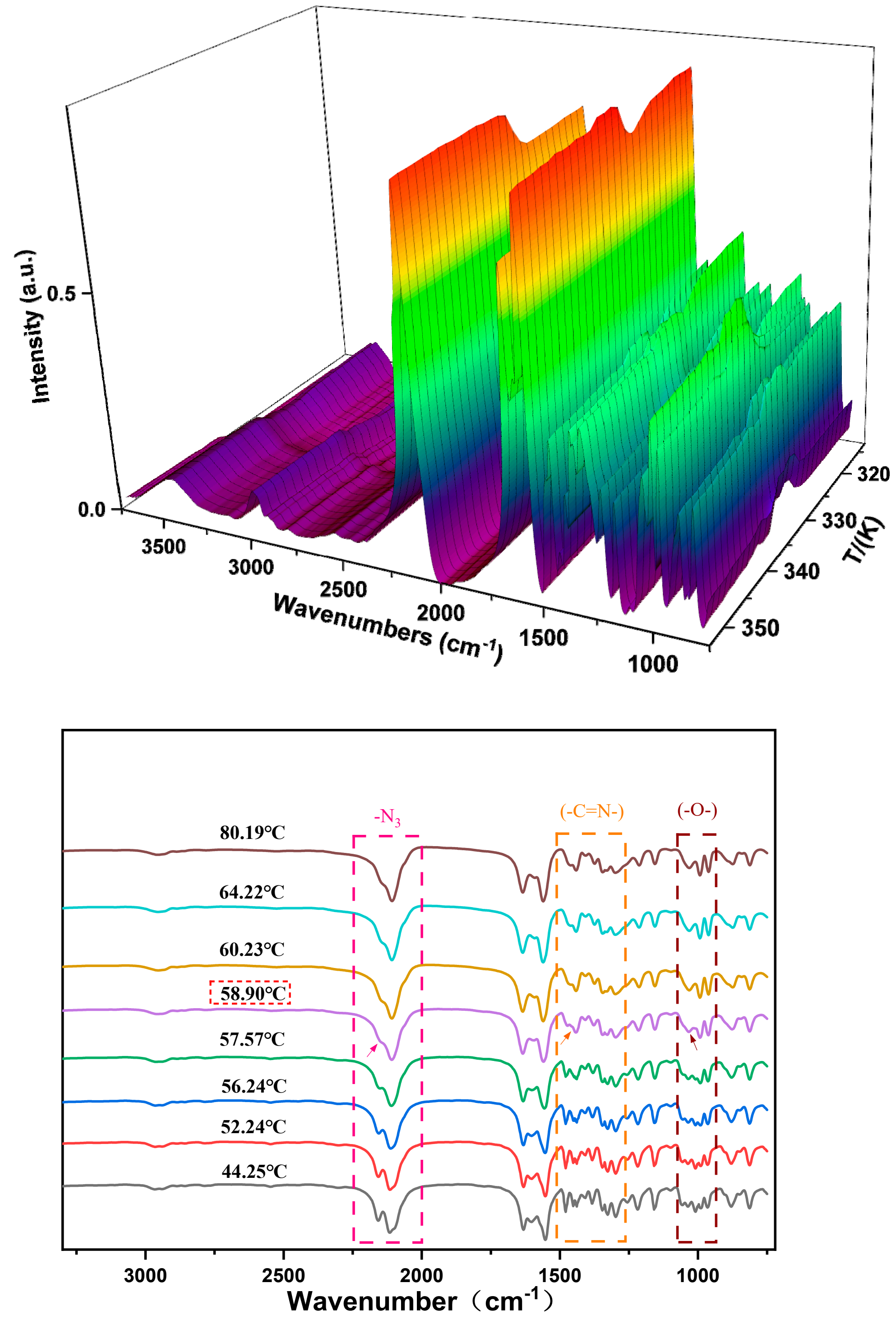

Figure S3. In-situ FTIR spectrum of the melting process of DAeTF.

6. FTIR and NMR spectra of 2-azidoethanol

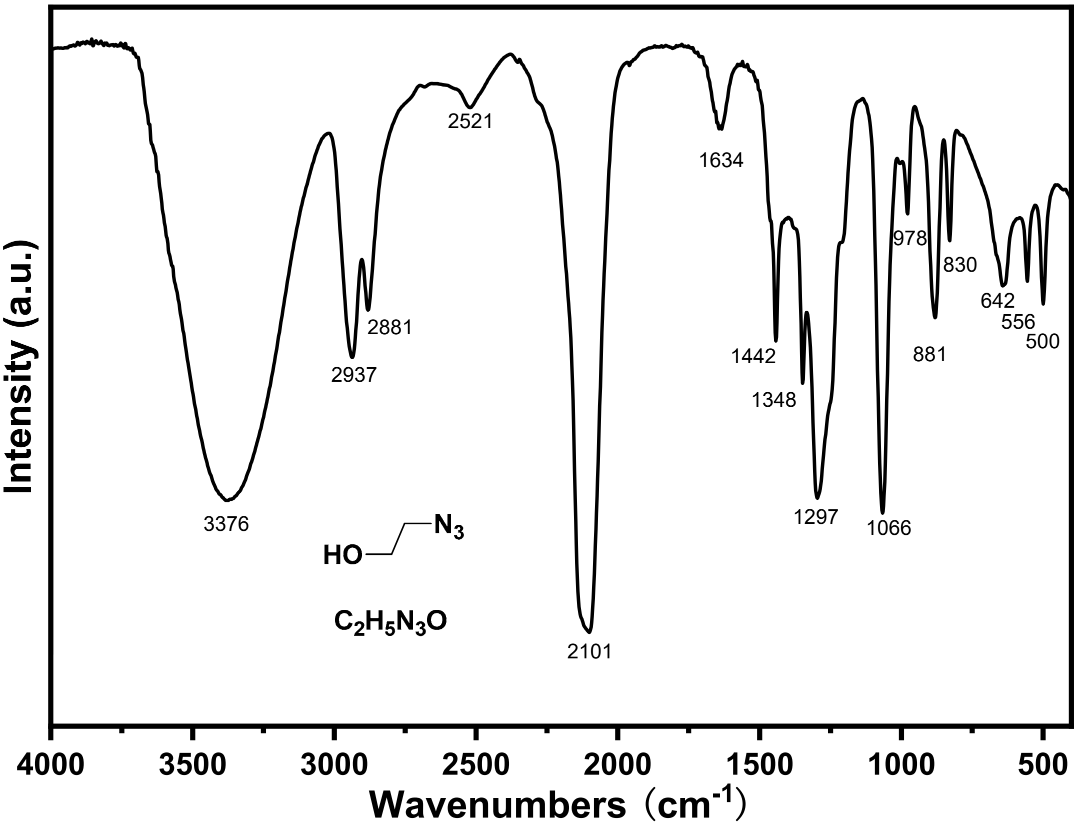

Figure S4. FTIR spectrum of 2-azidoethanol.

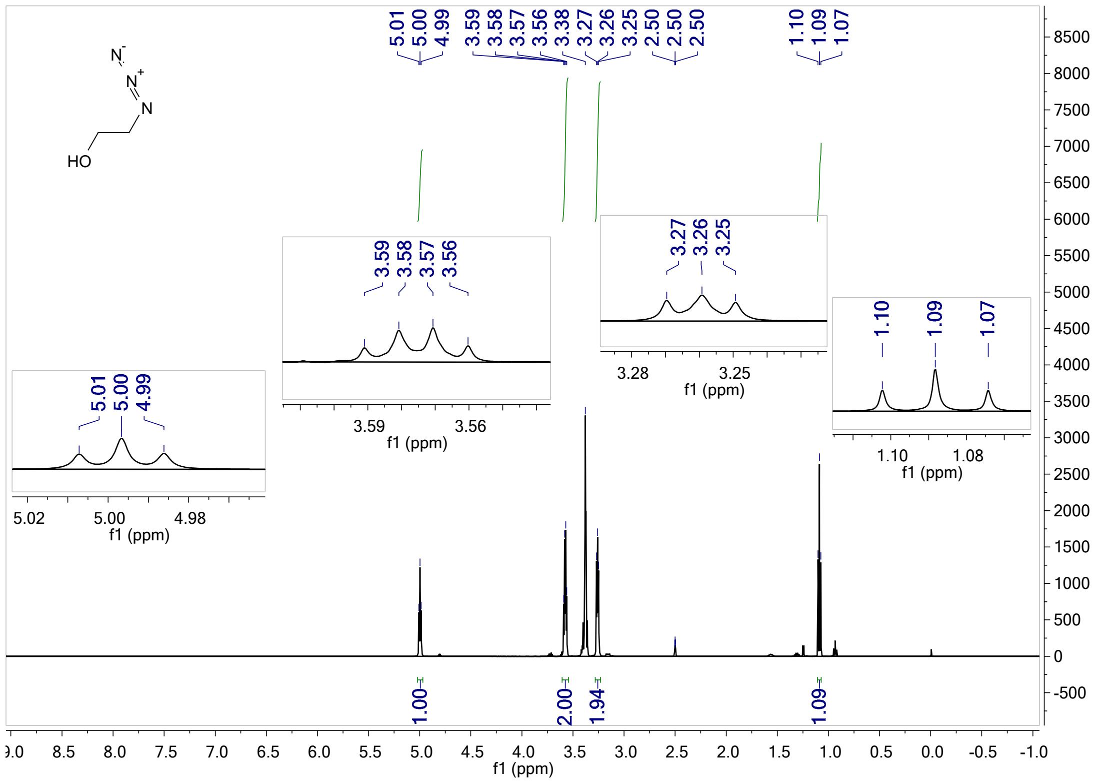

Figure S5. ^{1}H-NMR spectrum (500 MHz, DMSO-d_6, 298K) of 2-azidoethanol.

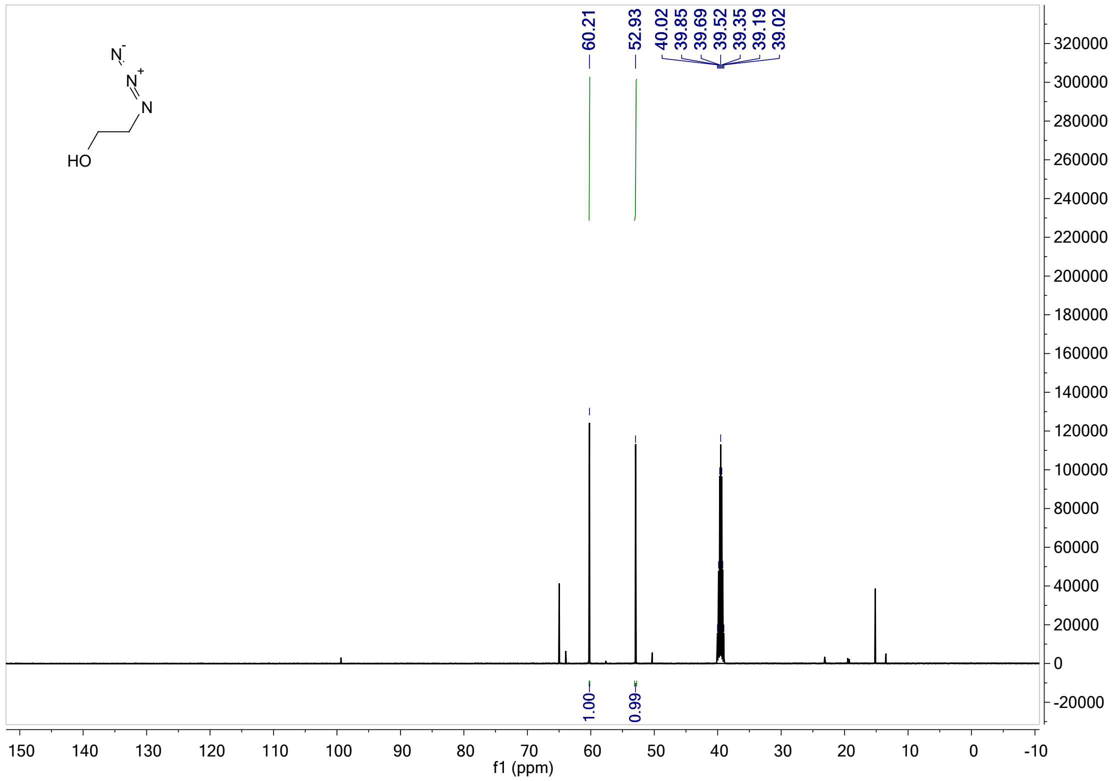

Figure S6. ^{13}C-NMR spectrum (125 MHz, DMSO-d_6, 298K) of 2-azidoethanol.

7. FTIR and NMR spectra of NAeTF, AeNTF

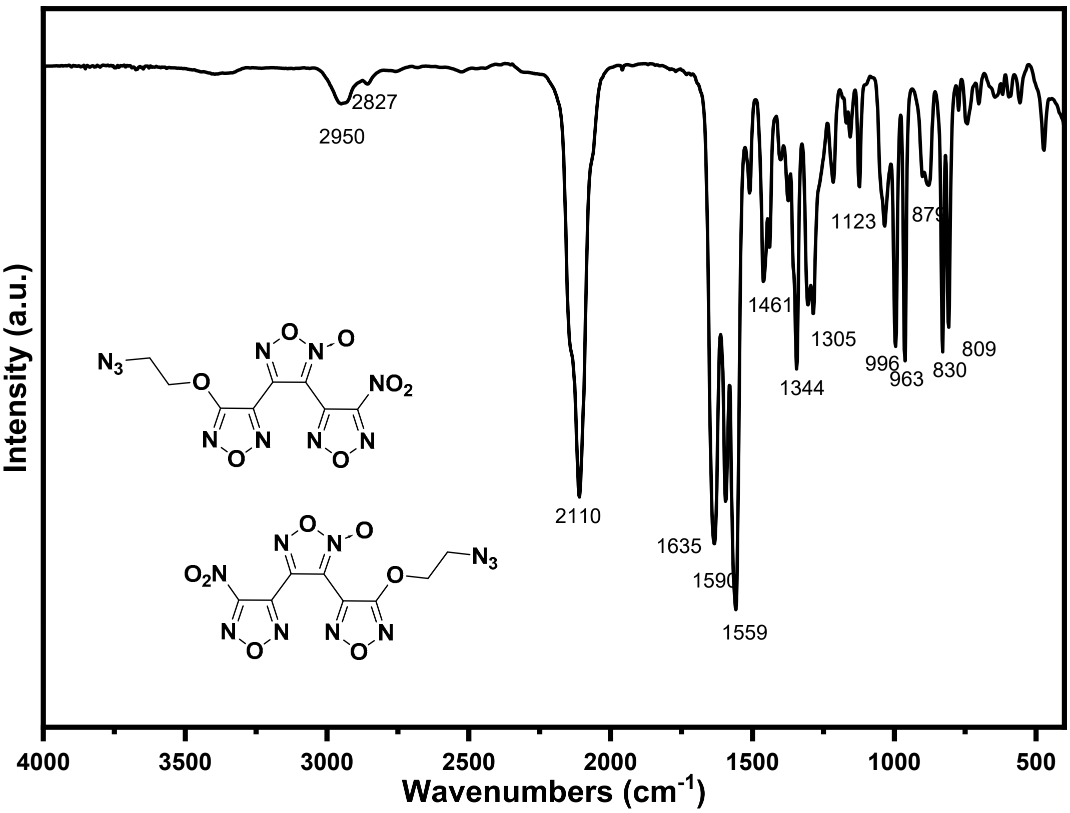

Figure S7. FTIR spectrum of the NAeTF and AeNTF mixed liquid.

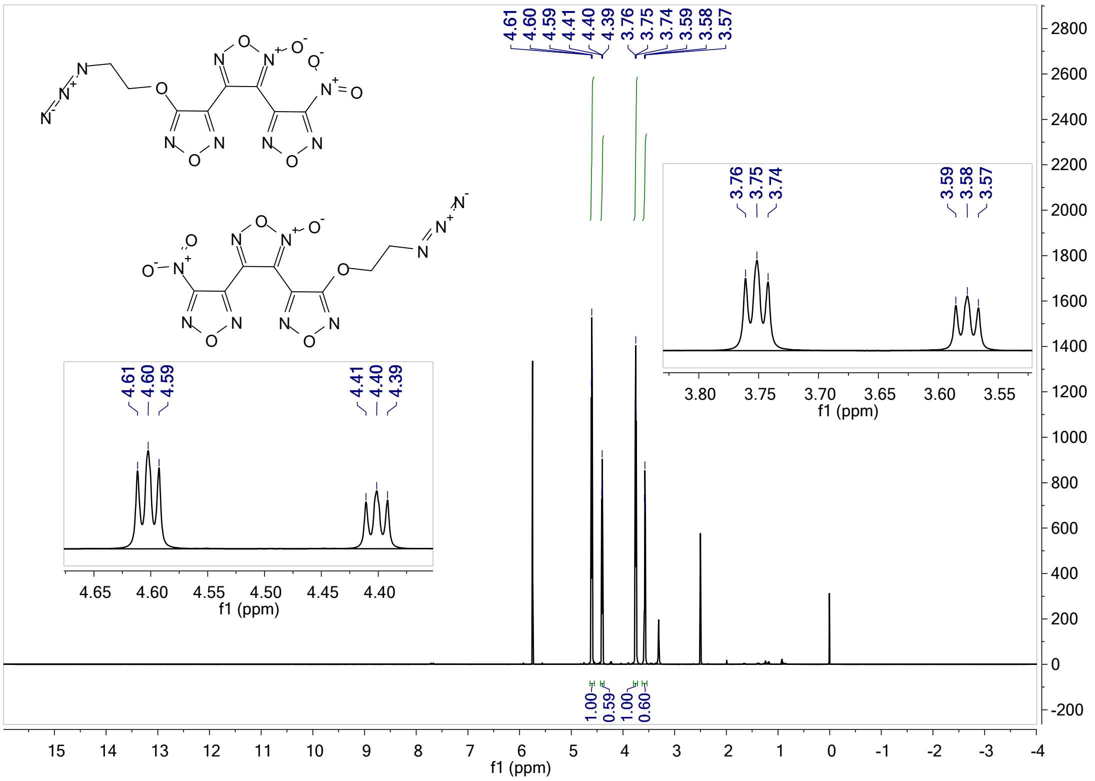

Figure S8. ^{1}H-NMR spectrum (500 MHz, DMSO-d_6, 298K)of the NAeTF and AeNTF Mixed Liquid.

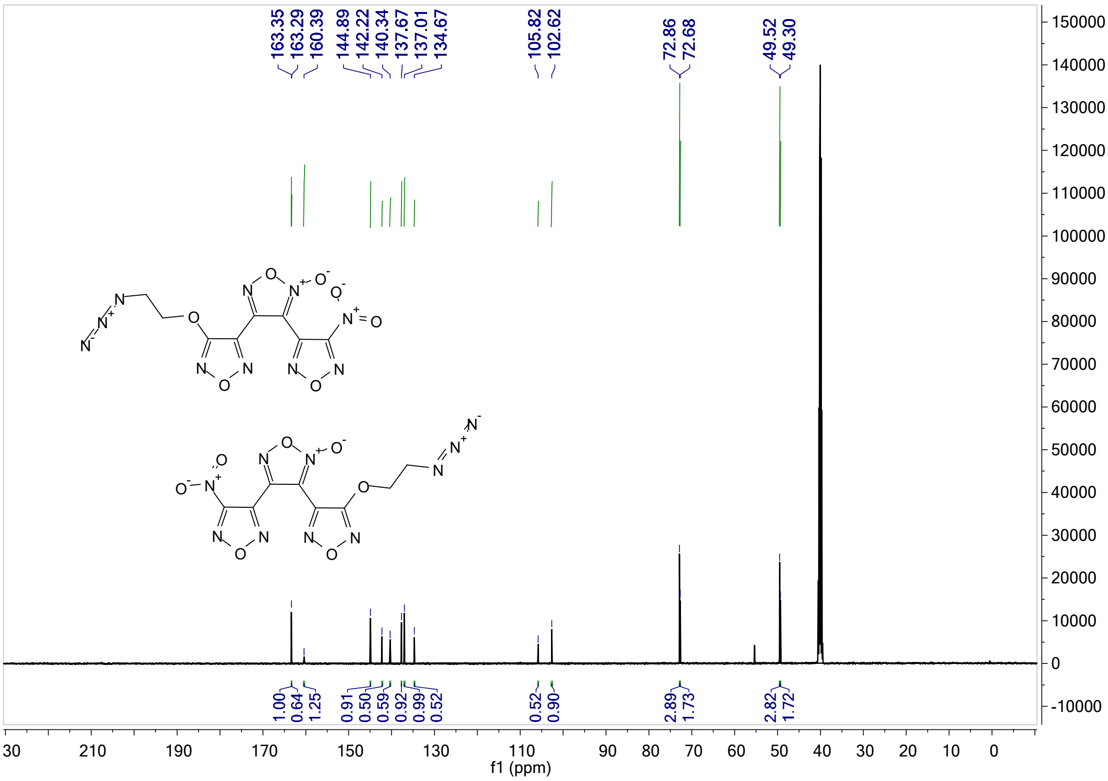

Figure S9. ^{13}C-NMR spectrum (125 MHz, DMSO-d_6, 298K) of the NAeTF and AeNTF Mixed Liquid.

8. FTIR, NMR, and mass spectra of DAeTF

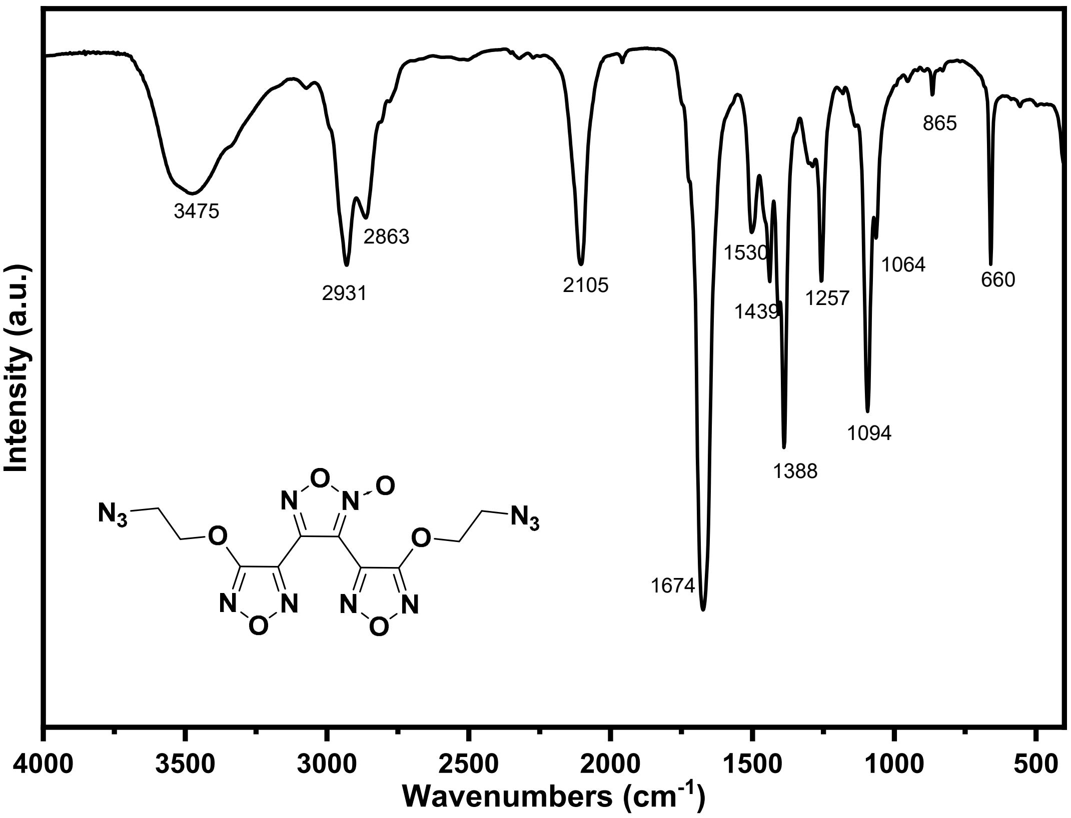

Figure S10. FTIR spectrum of DAeTF.

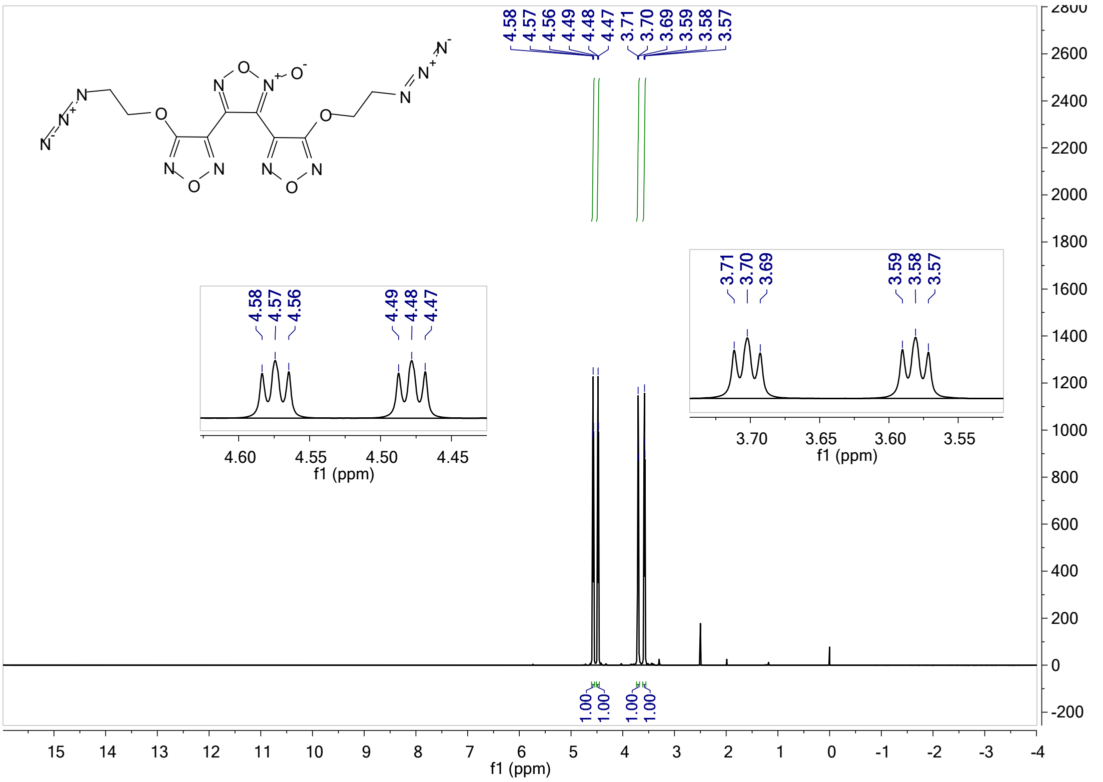

Figure S11. ^{1}H-NMR spectrum (500 MHz, DMSO-d_6, 298K) of DAeTF.

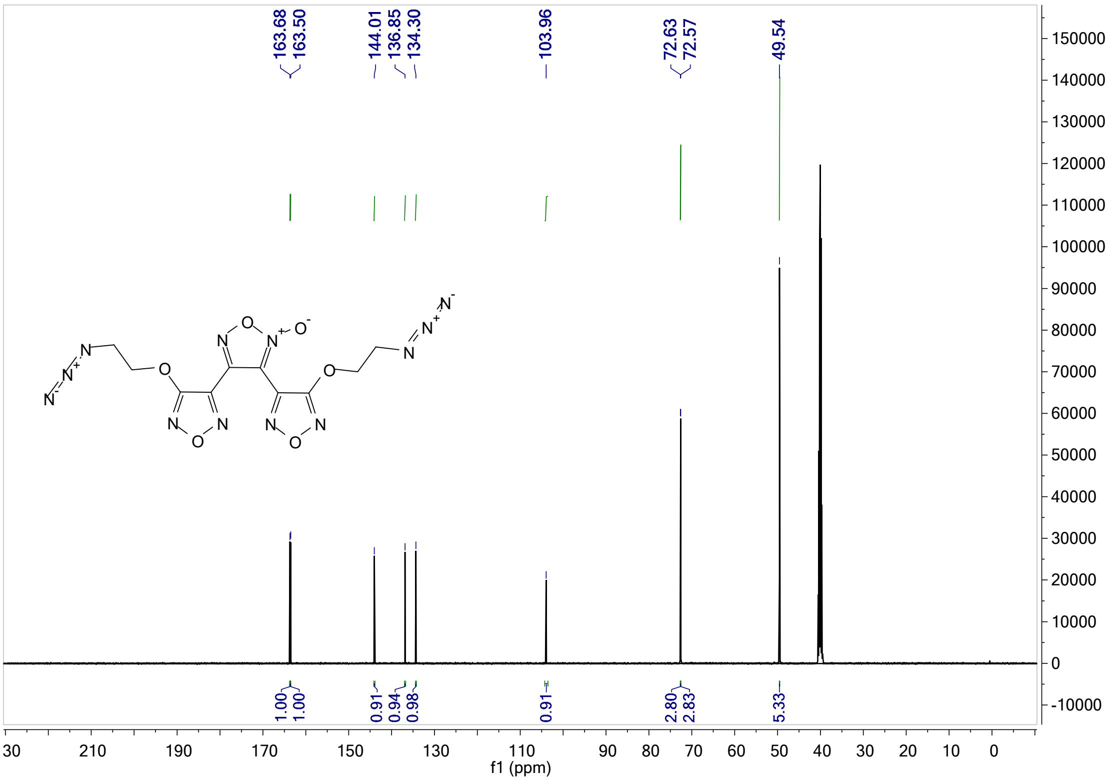

Figure S12. ^{13}C-NMR spectrum (125 MHz, DMSO-d_6, 298K) of DAeTF.

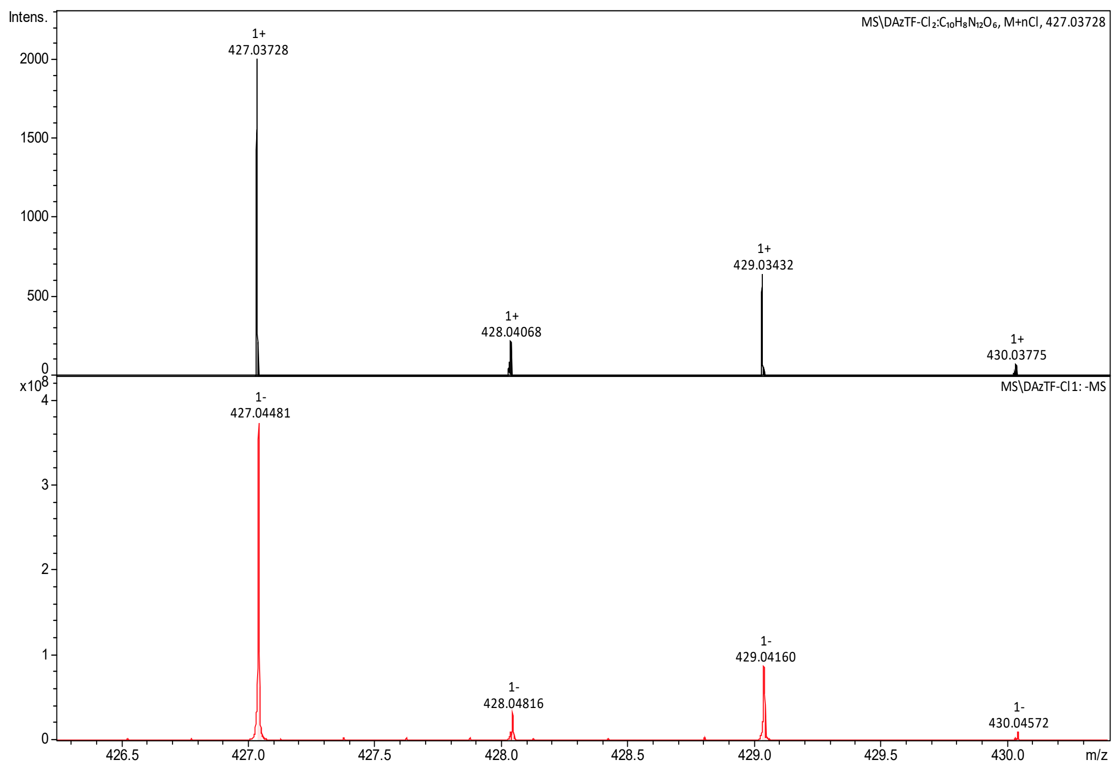

Figure S13. Mass spectrum of DAeTF.

References

[1] Luo, Y.; Ju, R.; Li, B.; Meng, J.; Wang, X. Thermal Decomposition and Solidification Characteristics of BFFO. Crystals 2023, 13, 802. https://doi.org/10.3390/cryst13050802
